# A Luminex Approach to Develop an Anti-Tumor-Associated Antigen Autoantibody Panel for the Detection of Prostate Cancer in Racially/Ethnically Diverse Populations

**DOI:** 10.3390/cancers15164064

**Published:** 2023-08-11

**Authors:** Cuipeng Qiu, Xiao Wang, Serina A. Batson, Bofei Wang, Carlos A. Casiano, Giulio Francia, Jian-Ying Zhang

**Affiliations:** 1 Department of Biological Sciences & NIH-Sponsored Border Biomedical Research Center, The University of Texas at El Paso, El Paso, TX 79968, USA; cqiu3@utep.edu (C.Q.); xwang6@utep.edu (X.W.); sabatson2@miners.utep.edu (S.A.B.); bwang2@miners.utep.edu (B.W.); 2 Center for Health Disparities and Molecular Medicine, Department of Basic Sciences, Loma Linda University School of Medicine, Loma Linda, CA 92354, USA; ccasiano@llu.edu

**Keywords:** prostate cancer, autoantibodies, tumor-associated antigens (TAAs), biomarkers, Luminex assay

## Abstract

**Simple Summary:**

This study explored the use of Luminex technology to detect fourteen autoantibodies associated with cancers and optimize an autoantibody panel for the detection of prostate cancer. Twelve autoantibodies were found at significantly higher frequencies in the prostate cancer group. One specific autoantibody, anti-HSP60, was further validated using an enzyme-linked immunosorbent assay with a larger sample size. The findings from both the Luminex and ELISA methods were consistent. We then combined three autoantibodies (p16, IMP2, and HSP60) to create a panel that achieved a high accuracy rate in detecting prostate cancer, with a sensitivity of 71.4% and specificity of 95.8%. This panel was evaluated in patients from different race/ethnicity backgrounds, and it showed the best performance in differentiating Hispanic American prostate cancer patients from healthy individuals. Overall, this study presents a promising approach for the detection of prostate cancer using a panel of specific autoantibodies, offering a potential tool for high-throughput screening.

**Abstract:**

(1) Background: Autoantibodies to tumor-associated antigens (TAAs) have emerged as promising cancer biomarkers. Luminex technology offers a powerful approach for the simultaneous detection of multiple anti-TAA autoantibodies. (2) Methods: We aimed to utilize Luminex technology to evaluate and optimize a panel of anti-TAAs autoantibodies for detecting prostate cancer (PCa), which included autoantibodies to fourteen TAAs. A total of 163 serum samples (91 PCa, 72 normal controls) were screened to determine the levels of the autoantibodies using the Luminex assay. (3) Results: Twelve autoantibodies exhibited significantly high frequencies ranging from 19.8% to 51.6% in the PCa group. Receiver operating characteristic (ROC) curve analysis revealed area under the curve (AUC) values ranging from 0.609 to 0.868 for the twelve autoantibodies individually. We further confirmed the performance of the HSP60 autoantibody by using an enzyme-linked immunosorbent assay (ELISA) in a larger sample comprising 200 PCa sera, 20 benign prostatic hyperplasia (BPH) sera, and 137 normal control sera. The results obtained from the Luminex assay were consistent with the ELISA findings. We developed a panel consisting of three autoantibodies (p16, IMP2, and HSP60) which achieved an impressive AUC of 0.910 with a sensitivity of 71.4% and a specificity of 95.8%. The panel was also evaluated in PCa patients from different races/ethnicities with the best performance observed in distinguishing the Hispanic American patients with PCa from normal controls. (4) Conclusions: We developed an anti-TAA autoantibody panel for the detection of PCa that exhibits promising performance. This panel holds significant potential as a high-throughput tool to facilitate PCa detection.

## 1. Introduction

Prostate cancer (PCa) is the most commonly diagnosed cancer and the second leading cause of cancer death among men worldwide [1]. PCa is asymptomatic in the early stage of the disease and is characterized by a large subset of the indolent cancer type [2]. Despite the development of effective screening methods, such as the prostate-specific antigen (PSA) test, the specificity and sensitivity of current diagnostic tests for PCa are still suboptimal, leading to high rates of false-positive and false-negative results [3]. The identification of new biomarkers for the detection of PCa is therefore an area of active research, and autoantibodies against tumor-associated antigens (TAAs) have emerged as promising diagnostic markers for cancer. Autoantibodies to TAAs have been considered as reporters of early carcinogenesis and indicators of cancer prognosis [4]. These autoantibodies are abundant, detectable in early stages, and stable in the patient’s circulating blood [5]. While the sensitivity of a single autoantibody is usually low, a panel of multiple autoantibodies could reach much higher sensitivity [6]. An optimized multiplex autoantibody panel to TAAs can improve the potential of using autoantibodies as biomarkers, and it can serve as a relatively simple tool to evaluate large numbers of patient samples for cancer detection [7].

Comprehensive serological screening can identify blood-based biomarkers to facilitate cancer detection. Our group has previously utilized serological proteome analysis (SERPA), Western blotting, and ELISA to identify and evaluate some potential anti-TAA autoantibodies for cancer detection [5]. We have reported that the levels of autoantibodies to p53, p16, IMP2/p62, IMP3, BIRC5/Survivin, and HSP60 were elevated in the sera from patients with hepatocellular carcinoma [8,9,10], breast cancer [11], and lung cancer [12]. We have also reported significantly higher levels of autoantibodies to p53, ENO1, and BIRC5 in patients with PCa compared to healthy controls [13,14]. In particular, autoantibodies to p53, p16, and p62 were widely detected in multiple tumors [15,16,17,18]. Additionally, other autoantibodies targeting SOX2, HIF1-α, NY-ESO-1/CTAG1B, MUC1, Her2, GAL1, and GAL3 have been frequently identified in cancer patients [16,18,19,20,21,22]. Given the high number of human cancers in which these autoantibodies are expressed, we have termed these autoantibodies “common cancer-associated autoantibodies (CAAs)”. Although some CAAs have been detected in PCa, no previous studies have performed multiplex profiling of these CAAs in PCa patients. Moreover, there is a paucity of data regarding potential racial variations in common CAAs among PCa patients of different ethnic backgrounds.

In recent years, Luminex bead technology has been increasingly used for the detection of proteins, including autoantibodies, in patients’ serum samples, as it offers high throughput and multiplexing capabilities, enabling the simultaneous detection of multiple autoantibodies in a single assay [23,24]. Importantly, it only requires a limited amount of serum sample (roughly 25 μL of diluted sample per multiplex run). This multiplex bead-based immunoassay can detect up to 100 analytes simultaneously. In this study, we sought to develop and optimize an optimal panel of anti-TAA autoantibodies for the detection of PCa using the Luminex technology, with the goal of improving the accuracy of PCa diagnosis and facilitating earlier intervention and treatment. Fourteen TAAs (p53, p16, IMP2, IMP3, SOX2, BIRC5, HIF1-α, HSP60, ENO1, CTAG1B, MUC1, Her2, GAL1, and GAL3) that have been commonly reported to be targets of autoantibodies in various cancer types were used to profile their corresponding autoantibodies in patients with PCa. We further validated our findings from the Luminex assay using ELISA that was subsequently performed on a larger group of patients. Additionally, we analyzed the performance of the CAA panel among PCa patients from three different racial/ethnic groups: African Americans (AA), Caucasian Americans (CC), and Hispanic Americans (HA). We also explored the potential association of these autoantibodies with surgery by profiling for common CAAs in sera from PCa patients before and after surgery. Overall, our findings demonstrated the association of specific CAAs with PCa and highlighted the potential of an anti-TAA autoantibody panel as a diagnostic tool with improved sensitivity and specificity for PCa detection.

## 2. Materials and Methods

### 2.1. Serum Samples

In this study, a total of 357 serum samples were analyzed, consisting of 200 sera from patients with PCa, 20 sera from patients with benign prostatic hyperplasia (BPH), and 137 normal control sera. The samples were obtained from the serum bank at the Cancer Autoimmunity and Epidemiology Research Laboratory at UTEP (The University of Texas at El Paso), Texas. Due to limited budget, we reduced the sample size for the Luminex assay to save on costs. Also, we tried to include a diverse range of race and ethnicity among the PCa sera. From these samples, we randomly selected 91 PCa sera (the patients comprised 20 AA, 51 CC, and 20 HA) and 72 normal control sera for Luminex assay testing. Among the 91 PCa patients, 12 patients had sera before and after surgery (3 to 6 months after surgery). All the 357 samples were used for the ELISA confirmatory test. Normal control sera were obtained from individuals who showed no obvious signs of malignancy. To comply with regulations regarding studies involving human subjects, the patients’ names and identification numbers were blinded to the investigators. Furthermore, partial clinical information was unavailable for some serum samples.

### 2.2. Luminex Immunoassay

MILLIPLEX^®^ Human Cancer Autoantibody Magnetic Bead Panel kits were purchased from Millipore Sigma (Burlington, MA, USA). The magnetic bead panel detects autoantibodies to 14 TAAs (p53, p16, IMP2, IMP3, SOX2, BIRC5, HIF1-α, HSP60, ENO1, CTAG1B, MUC1, Her2, GAL1, and GAL3) via specifically prepared magnetic beads. The immunoassay was conducted by following user guidelines. In brief, serum samples were diluted at 1:100 in Assay Buffer and incubated together with TAA-coated beads in 96-well plates overnight at 4 °C, followed by washing the plates 3 times with Wash Buffer. PE-IgG Conjugate was then added to each well, followed by incubation for 90 min at room temperature. After washing 3 times with Wash Buffer, Sheath Fluid PLUS was added to each well and plates were read on the Luminex^®^ platform and analyzed with xPONENT software (SN: MAGPX15334703, Version: 4.2). median fluorescent intensity (MFI) data were obtained for further analysis.

### 2.3. Enzyme-Linked Immunosorbent Assay (ELISA)

For this study, the full-length recombinant protein HSP60 was obtained from Abcam (Boston, MA, USA). The protein had a purity of more than 90% as determined through SDS-PAGE analysis. The ELISA assay procedure followed the methodology outlined in a previous study [25]. Briefly, HSP60 protein was diluted in phosphate-buffered saline (PBS) (Thermo Scientific, Ward Hill, MA, USA) to a final concentration of 1 μg/mL. This diluted protein solution (100 μL/well) was then coated onto 96-well plates and incubated overnight at 4 °C. The plates were coated with a gelatin post-coating solution (200 μL/well) and incubated for 2 h at room temperature. Subsequently, the plates were washed three times with PBS containing 0.05% Tween-20 (PBST). Serum samples were diluted 1:200 and added to the plates as primary antibodies. The plates were then incubated for 1 h at 37 °C in a water bath. Following incubation, the plates were washed five times with PBST. To detect the bound antibodies, horseradish peroxidase (HRP)-conjugated goat anti-human IgG was added to the plates at a 1:3000 dilution (100 μL/well). The plates were incubated for 1 h at 37 °C in a water bath, followed by five washes with PBST. For the colorimetric detection of the antibodies, a substrate solution using 2,2′-azino-bis (3-ethyl-benzothiazoline-6-sulfonic acid) (ABTS) was employed. The optical density (OD) values were measured at a wavelength of 405 nm.

### 2.4. Statistical Analysis

Nonparametric statistical tests (Kruskal–Wallis H test and Mann–Whitney U test) were used to assess the differences in the distribution of the autoantibody levels among various groups. The Wilcoxon test was utilized to compare the difference between patient sera before and after surgery. The chi-square test was employed to evaluate the difference in autoantibody frequency between two groups. A two-sided significance level of 0.05 was used, and the *p*-values were adjusted using the Bonferroni correction for multiple tests. The receiver operating characteristic (ROC) curve analysis was applied to evaluate the diagnostic potential of each autoantibody, and help assess the sensitivity, specificity, Youden index (YI), positive predictive value (PPV), negative predictive value (NPV), and accuracy of each autoantibody. Binary logistic regression (forward conditional method) was conducted for the development of an optimal CAA panel. To establish cut-off values for defining a positive result from normal controls (NC), a specificity of more than 95.0% was considered.

## 3. Results

### 3.1. Higher Frequencies of Anti-TAA Autoantibodies in PCa Patients Compared to Normal Controls

**Figure 1:**
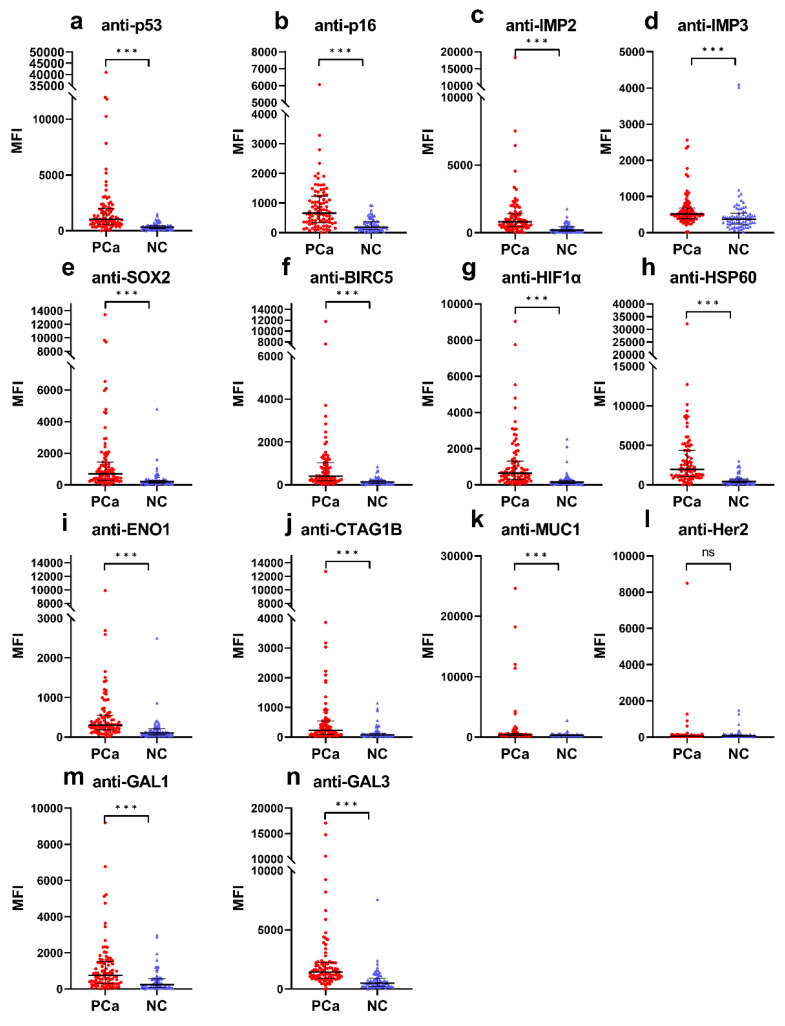
Median fluorescent intensity (MFI) values of 14 autoantibodies (**a**–**n**) in prostate cancer (PCa) group and normal control (NC) group. The MFI values are shown in scatter plots with median and quartiles. ***, *p* > 0.001; ns, not significant.

Since the selected 14 common CAAs have been detected in various human cancers, we first sought to evaluate whether these autoantibodies are also associated with PCa. Of the 14 CAAs assessed with the Luminex assay, 13 (anti-p53, anti-p16, anti-IMP2, anti-IMP3, anti-SOX2, anti-BIRC5, anti-HIF1-α, anti-HSP60, anti-ENO1, anti-CTAG1B, anti-MUC1, anti-GAL1, and anti-GAL3) showed significantly higher MFI values in the PCa group than in the NC group (Figure 1). After we set the cut-off value based on NC group (specificity > 95.0%) to maintain a false positive rate (FPR) of less than 5.0% by limiting the number of control subjects with positive results, the anti-IMP3 autoantibody had a higher cut-off value because it had an MFI value higher than 4000 in two NC samples (Figure 1d), thus resulting in no significant difference in frequency between the two groups (Table 1). Except for the autoantibodies to IMP3 (8.8% frequency) and Her2 (4.4% frequency), the frequencies (at specificity > 95.0%) of the other 12 anti-TAA autoantibodies were significantly increased (*p* < 0.001) in the PCa group compared to the NC group (Table 1). The frequencies of these 12 autoantibodies ranged from 19.8% to 51.6% at 95.8% specificity for normal controls. Over 40 of the 91 PCa patients showed positive autoantibody reactions to p53, p16, IMP2, and HSP60. The autoantibody to HSP60 had the highest median MFI value (19,840.5) in the PCa group while the autoantibody to p53 exhibited the highest frequency of 51.6% (47 of 91) in the PCa group (Figure 1 and Table 1).

### 3.2. Performance of Anti-TAA Autoantibodies in Distinguishing PCa from Normal Controls

**Figure 2:**
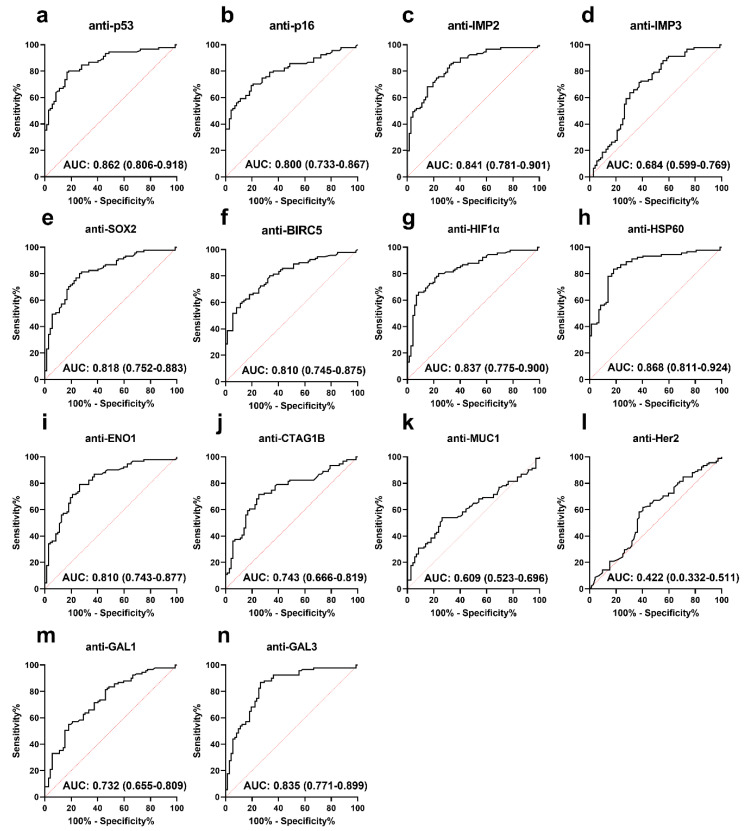
ROC analysis of 14 autoantibodies (**a**–**n**) in the identification of PCa from normal controls. AUC, area under the curve. The AUC and its 95% confidence interval are shown in the figure for each autoantibody.

To investigate the performance of the anti-TAA autoantibodies in the identification of PCa compared to NC, we performed an ROC analysis, which can comprehensively reflect the diagnostic value of each autoantibody. Twelve autoantibodies showed low to moderate PCa identification performance with the AUC ranging between 0.609 and 0.868 (Figure 2). As shown in Figure 2, the autoantibodies to HSP60 and p53 exhibited a relatively higher AUC of 0.868 and 0.862 to distinguish PCa from NC. Notably, nine autoantibodies (anti-p53, anti-p16, anti-IMP2, anti-SOX2, anti-BIRC5, anti-HIF1-α, anti-HSP60, anti-ENO1, and anti-GAL3) showed a good diagnostic value with AUC ≥ 0.800 and accuracy > 60%. (Figure 2 and Table 1).

### 3.3. ELISA Validation of Anti-HSP60 Autoantibody in a Large Sample

**Figure 3:**
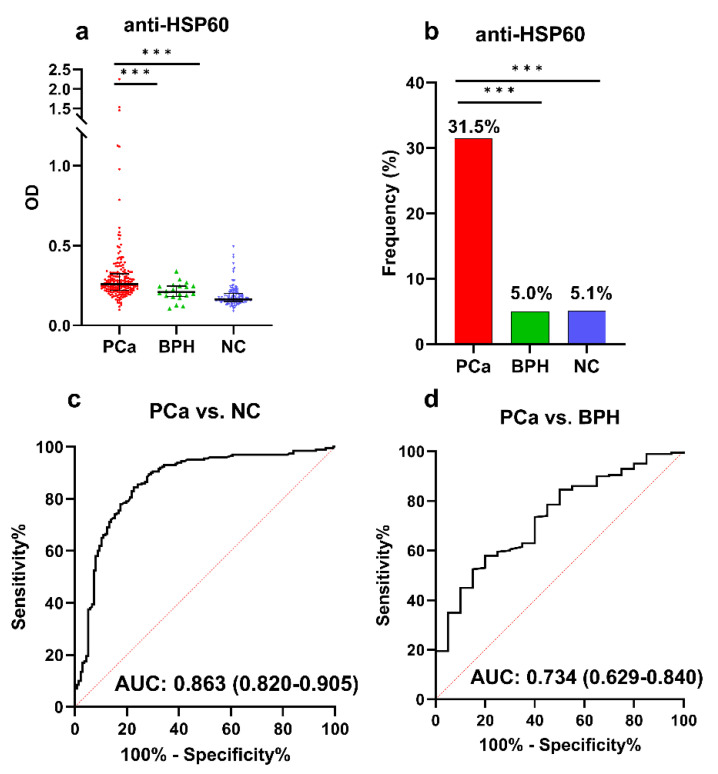
Performance of the autoantibody to HSP60 in the detection of PCa in ELISA. (**a**) OD values of autoantibody to HSP60 in PCa, BPH, and NC; (**b**) prevalence of autoantibody to HSP60 among the three groups; (**c**) performance of autoantibody to HSP60 for identifying PCa from NC; (**d**) performance of autoantibody to HSP60 for identifying PCa from BPH. PCa, prostate cancer; NC, normal controls; BPH, benign prostatic hyperplasia; OD, optimal density; Se, sensitivity; Sp: Specificity. ***, *p* > 0.001. The AUC and its 95% confidence interval are shown in the figure.

Due to the limited sample size (91 PCa and 72 NC) in the Luminex assay, we further validated the performance of the anti-HSP60 autoantibody using ELISA in a larger group of patients. A total of 357 serum samples including 200 PCa, 137 NC, and 20 BPH were used to evaluate the performance of the anti-HSP60 autoantibody with ELISA. As shown in Figure 3a, the serum levels of the anti-HSP60 autoantibody were significantly higher in patients with PCa than in the NC or BPH groups (*p* < 0.001). The ELISA results revealed that 63 of 200 patients (31.5%) were positive for the anti-HSP60 autoantibody at a specificity of 94.9% (Figure 3b). The autoantibody to HSP60 showed an AUC of 0.863 and 0.734 to distinguish PCa from NC and BPH, respectively (Figure 3c,d). The results from ELISA were consistent with Luminex assay findings.

### 3.4. Developing an Optimal Anti-TAA Autoantibody Panel for PCa Identification

**Figure 4:**
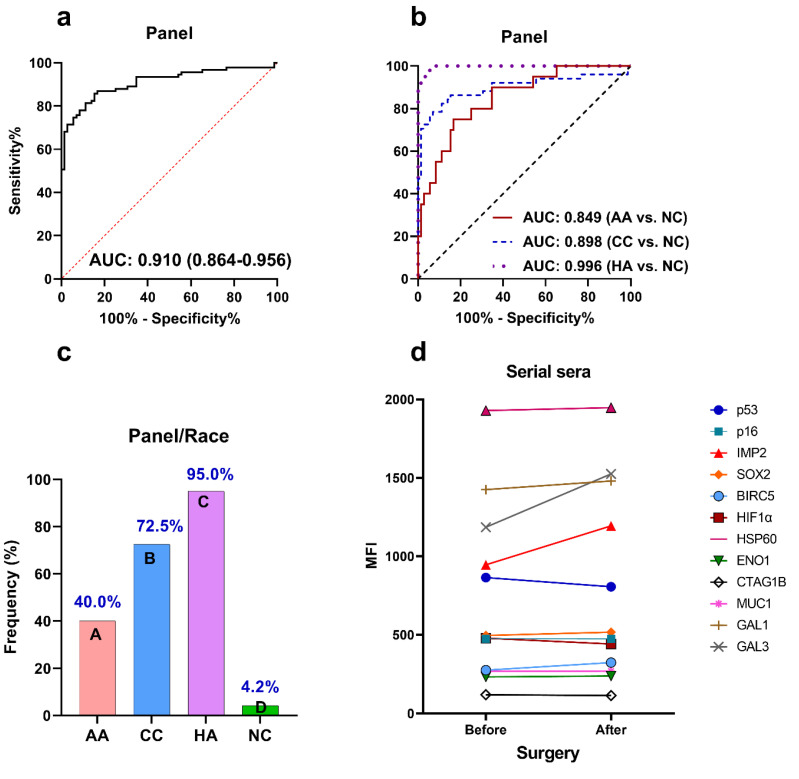
Performance of the anti-TAA autoantibody panel (p16, IMP2, and HSP60) in the identification of PCa and the levels of 12 anti-TAA autoantibodies in serial sera before and after surgery. (**a**) AUC with 95% confidence interval of the anti-TAA autoantibody panel for identifying PCa from NC; (**b**) performance of the anti-TAA autoantibody panel for identifying PCa patients of three different races from NC (red curve: AA vs. NC, blue dotted curve: CC vs. NC, purple dotted curve: HA vs. NC); (**c**) frequencies of the anti-TAA autoantibody panel in PCa patients of three different races (different letters A–D above the bars mean a significant difference was observed); (**d**) the autoantibody trend change in 12 PCa patients before and after surgery (3 to 6 months after radical prostatectomy). The points at both ends of the line represent the MFI value of each anti-TAA autoantibodies. PCa, prostate cancer; NC, normal controls; Se, sensitivity; Sp: specificity; AA, African Americans; CC, Caucasians; HA, Hispanic Americans.

Since the frequency of a single autoantibody against an individual TAA is typically low in most cancer types, the antibody response to a multiplexed panel of TAAs could improve the frequency of autoantibodies for identifying cancer patients. However, considering cost-effectiveness, we tried to narrow the number from 12 anti-TAA autoantibodies down to the ideal number without reducing the performance of the panel. In this study, logistic regression was applied to develop an optimal anti-TAA autoantibody panel with the combination of multiple TAAs. We finally narrowed the panel to three TAAs (p16, IMP2, and HSP60). The logistic regression model was developed as follows: Logit (P = PCa) = −3.057 + 0.002 × p16 + 0.002 × IMP2 + 0.001 × HSP60. The panel exhibited an AUC of 0.910, a sensitivity of 71.4%, a specificity of 95.8%, and a PPV of 95.6%, which significantly enhanced the detection of PCa compared to a single autoantibody (Table 2 and Figure 4a).

In addition, we analyzed the performance of the TAA panel for autoantibody detection among PCa patients from different race/ethnicity backgrounds, including 20 AA, 51 CC, and 20 HA. We found that the panel showed the best performance in distinguishing HA PCa patients from NC, with an AUC of 0.996 (Figure 4b). There were 19 of 20 (95.0%) HA patients with PCa that had a positive reaction of autoantibodies to the TAA panel (Table 2 and Figure 4c). In distinguishing CC PCa patients from NC, the TAA panel exhibited an AUC of 0.898 and a frequency of 72.5% (37 of 51). Also, the panel could distinguish 8 of 20 (40.0%) AA PCa from NC with an AUC of 0.849 (Figure 4b,c).

### 3.5. Changes in Autoantibodies in Patients with PCa before and after Surgery

To identify potential surgery-associated autoantibodies, we tested the 12 anti-TAA autoantibodies with higher frequency in PCa against sera from 12 patients with PCa who had serum samples available before and after surgery (radical prostatectomy). Sera were drawn 3 to 6 months (average 4 months) after surgery. The results showed that most of these 12 autoantibodies were stable after surgery, as evidenced by their lack of change in their MFI (Figure 4d). However, autoantibodies to GAL3 and IMP2 were elevated after surgery given their increased MFIs. Interestingly, the MFI for anti-P53 autoantibodies was slightly decreased after surgery (Figure 4d). These findings suggest that the levels of autoantibodies to p53, IMP2, and GAL3 might be influenced by surgery.

## 4. Discussion

The present study focused on the evaluation and optimization of an anti-TAA autoantibody panel for the detection of PCa using Luminex technology. The use of anti-TAA autoantibodies as biomarkers for cancer detection has gained increasing attention in recent years, as autoantibodies are produced early in cancer development and have the potential to offer higher sensitivity than traditional biomarkers [26]. In particular, autoantibodies against TAAs have emerged as promising diagnostic markers for cancer [27]. Leveraging the power of Luminex technology, we simultaneously detected fourteen common CAAs in the sera of patients with PCa. Our findings demonstrated that twelve out of these CAAs exhibited significantly high frequencies in the PCa group, ranging from 19.8% to 51.6%, while maintaining a specificity of 95.8%. These results indicated that CAAs have the potential to serve as promising biomarkers for PCa detection. The receiver operating characteristic (ROC) curve analysis further confirmed the diagnostic potential of the individual autoantibodies, as indicated by the area under the curve (AUC) values ranging from 0.609 to 0.868.

High frequencies of positive autoantibody reactions and high AUC (AUC > 0.860) were observed for autoantibodies to p53 and HSP60, suggesting their potential as diagnostic biomarkers for PCa. Anti-p53 autoantibody has been demonstrated to be associated with various types of cancer including PCa [15,28,29]. Consistent with this, the Luminex assay detected an anti-p53 response in 51.6% (47/91) of PCa patients. HSP60, a member of the heat shock protein (HSP) family, is a promising biomarker for diagnosis and a potential treatment target for cancer [30]. It has been shown to be significantly elevated in human cancers [30,31]. Moreover, the anti-HSP60 autoantibody has been reported in the serum of cancer patients and can be used as a diagnostic biomarker [32]. For instance, the presence of this autoantibody has been reported in patients with hepatocellular carcinoma [8,33], breast cancer [16], ovarian cancer [34], and colorectal cancer [35]. In this study, we found 41.8% (38/91) of PCa patients tested positive for the anti-HSP60 autoantibody with a moderate diagnostic performance (AUC = 0.868). We further validated the performance of the anti-HSP60 autoantibody using ELISA and a larger sample size (357 serum samples). To explore the potential ability of the autoantibody to distinguish between PCa patients and benign patients, we also included patients with benign prostatic hyperplasia (BPH) in the ELISA testing. The ELISA detected 31.5% (63/200) of PCa patients who had positive autoantibody reactions to HSP60. It also showed a moderate diagnostic performance for PCa, with an AUC of 0.863. This autoantibody can distinguish between PCa and BPH with an AUC of 0.734. The consistent results obtained from the Luminex assay and ELISA strengthen the reliability and reproducibility of the Luminex technology for autoantibody detection [36]. These results further confirmed the significance of the anti-HSP60 autoantibody as a potential diagnostic marker for PCa.

The Luminex technology provides a powerful and efficient platform for the detection of autoantibodies. Particularly when adding clinical samples, of which limited quantities may sometimes be available, it is possible to maximize the information that can be obtained from limited serum/plasma using Luminex. Its ability to simultaneously measure multiple autoantibodies, coupled with high sensitivity, specificity, and cost-effectiveness, makes it a valuable tool in cancer research and clinical diagnostics [37]. Indeed, we recently reported such analysis from a clinical trial with 38 gastrointestinal cancer patients treated with metronomic chemotherapy [38]. Using this technology, we were able to identify 12 common CAAs that were detected at relatively higher frequencies in PCa compared to controls. Previous studies from our group and others have demonstrated that an optimized panel of autoantibodies targeting multiple TAAs could achieve a better detection performance [7,9,10,13,17,23,39,40]. To further optimize a cost-effective anti-TAA autoantibody panel that could improve detection frequency without compromising performance, we developed a limited panel comprising three anti-TAA autoantibodies (anti-p16, anti-IMP2, and anti-HSP60) based on logistic regression modeling. This panel demonstrated superior diagnostic performance, with an AUC of 0.910, sensitivity of 71.4%, specificity of 95.8%, and positive predictive value (PPV) of 95.6%. These results highlight the potential of a well-defined anti-TAA autoantibody panel as a powerful tool for PCa detection with high sensitivity and specificity, surpassing the performance of single autoantibodies. This panel could serve as a relatively simple and affordable tool in evaluating large numbers of patient serum samples in the screening of PCa patients.

Our group previously reported that there may be race/ethnicity-related differences in the presence or levels of specific CAAs among PCa patients [14,41]. To further explore this notion, we analyzed the performance of our anti-TAA autoantibody panel among PCa patients from different racial/ethnic groups. The panel showed excellent performance in identifying PCa in HA individuals compared to NC, with an AUC of 0.996. The high frequency of positive reactions among HA PCa patients further supports the panel’s effectiveness in this population. Although the panel exhibited slightly lower performance in identifying PCa among CC and AA individuals, it still demonstrated promising diagnostic capabilities. The biological basis for these racial/ethnic differences is not clear and deserves further investigation. However, it is important to note that the sample size of our study was relatively small. Therefore, it is crucial for future research to conduct large-scale studies involving a more diverse population with representation from various racial and ethnic backgrounds. This would help ensure the generalizability and robustness of the findings across diverse populations. Moreover, investigating the influence of race and ethnicity on anti-TAA autoantibody detection may shed additional light on racial/ethnic immunological determinants associated with cancer health disparities. This knowledge may contribute to the development of personalized medicine approaches that consider race and ethnicity in cancer diagnosis and treatment decision making.

We also explored the potential association of anti-TAA autoantibodies with surgery by analyzing sera collected from PCa patients before and after surgery to find potential biomarkers for disease monitoring. Most of the autoantibodies remained stable after surgery. A longitudinal study reported that the anti-TAA autoantibody profiles in serial serum samples from the same cancer patient were stable for 3 months after surgery, which was consistent with the current study [42]. Meanwhile, it is noteworthy that we observed interesting changes in the levels of three anti-TAA autoantibodies following surgery in PCa patients. Specifically, we noted elevated levels of autoantibodies to GAL3 and IMP2, whereas the autoantibody against p53 exhibited a slight decrease. The observed increase in autoantibodies to GAL3 and IMP2 after surgery suggests a potential association between the elicitation of these autoantibodies and the surgical intervention. Similarly, a previous study also found that autoantibodies to NPM1 in PCa patients significantly increased after surgery [41]. Further studies are needed to explore whether the elevated levels of anti-GAL3 and anti-IMP2 autoantibodies are a consequence of damage to tumor-adjacent tissue caused by the surgical intervention or if they indicate a systemic immune response triggered by the extracellular release of the overexpressed target antigens during or following the procedure. Conversely, the slight decrease in the autoantibody against p53 following surgery is an intriguing finding that warrants additional exploration. This decrease could reflect changes in the underlying disease state following surgical intervention. Studies have shown that the anti-p53 autoantibody could be a useful biomarker to monitor disease after therapy [43,44]. Given the strong association of anti-p53 autoantibodies with the presence of a tumor, it would be expected that these autoantibodies may decrease as the tumor is resected. Nevertheless, in order to comprehensively understand the clinical implications of these findings, future studies should incorporate larger patient and normal cohorts and analyze anti-TAA autoantibody profiles at multiple time points before and after surgery. Additionally, it would be valuable to assess the association between autoantibody dynamics and relevant clinical outcomes, such as disease recurrence or treatment response.

Future studies with larger sample sizes should be also performed to further confirm the performance of the anti-TAA autoantibody panel we developed and whether it could be used as supplementary tool for the diagnosis of PCa in combination with prostate-specific antigen (PSA). It is of interest to investigate the correlation between autoantibody intensity and patients’ survival in a larger sample with patients who have detailed clinical information. Moreover, it would be important to identify additional specific anti-TAA autoantibodies that selectively and significantly correlate with the presence of PCa compared to normal controls and patients with BPH or other cancer types. This could be achieved through high-throughput screening of thousands of patient sera against dozens of previously identified PCa-associated proteins or antigens.

## 5. Conclusions

In conclusion, this study presents a comprehensive evaluation and optimization of a well-defined anti-TAA autoantibody panel for the detection of PCa using Luminex technology. The panel, consisting of p16, IMP2, and HSP60 autoantibodies, exhibits promising performance with high diagnostic accuracy. The development of accurate and reliable biomarkers for the detection of PCa is crucial for improving patient outcomes and reducing the burden of this disease. While the present study provides valuable insights into the detection of anti-TAA autoantibodies for PCa diagnosis in diverse populations, the limited sample size and narrow racial/ethnic representation emphasize the need for future studies with larger and more diverse cohorts. By addressing these limitations, we can enhance the validity and clinical utility of the autoantibody panel and ensure its effectiveness across different racial groups. Our findings may contribute to the development of a more effective diagnostic tool for the detection of PCa and facilitate earlier intervention and treatment.

## Figures and Tables

**Table 1:** Performance of 14 autoantibodies in PCa patients and normal controls.

Autoantibody	Cut-Off (MFI)	Frequency (%)		*p*	Se (%)	Sp (%)	YI	PPV (%)	NPV (%)	Accuracy (%)
		PCa (*n* = 91)	NC (*n* = 72)							
anti-p53	994.3	47 (51.6)	3 (4.2)	<0.001	51.6	95.8	0.5	94.0	61.1	71.2
anti-p16	657.5	46 (50.5)	3 (4.2)	<0.001	50.5	95.8	0.5	93.9	60.5	70.6
anti-IMP2	834.5	44 (48.4)	3 (4.2)	<0.001	48.4	95.8	0.4	93.6	59.5	69.3
anti-IMP3	1086.5	8 (8.8)	3 (4.2)	0.198	8.8	95.8	0.0	72.7	45.4	47.2
anti-SOX2	926.8	35 (38.5)	3 (4.2)	<0.001	38.5	95.8	0.3	92.1	55.2	63.8
anti-BIRC5	624.0	35 (38.5)	3 (4.2)	<0.001	38.5	95.8	0.3	92.1	55.2	63.8
anti-HIF1α	660.3	44 (48.4)	3 (4.2)	<0.001	48.4	95.8	0.4	93.6	59.5	69.3
anti-HSP60	2317.8	38 (41.8)	3 (4.2)	<0.001	41.8	95.8	0.4	92.7	56.6	65.6
anti-ENO1	403.8	32 (35.2)	3 (4.2)	<0.001	35.2	95.8	0.3	91.4	53.9	62.0
anti-CTAG1B	570.5	21 (27.1)	3 (4.2)	<0.001	23.1	95.8	0.2	87.5	49.6	55.2
anti-MUC1	841.5	18 (19.8)	3 (4.2)	0.002	19.8	95.8	0.2	85.7	48.6	53.4
anti-Her2	489.8	4 (4.4)	3 (4.2)	0.628	4.4	95.8	0.0	57.1	44.2	44.8
anti-GAL1	1617.0	19 (20.9)	3 (4.2)	0.001	20.9	95.8	0.2	86.4	48.9	54.0
anti-GAL3	1882.5	30 (33.0)	3 (4.2)	<0.001	33.0	95.8	0.3	90.9	53.1	60.7

MFI, median fluorescent intensity; PCa, prostate cancer; NC, normal controls; Se, sensitivity; Sp, specificity; YI, Youden index; PPV, positive predictive value; NPV, negative predictive value. Cut-off values for defining a positive result from normal controls (NC) were set at a specificity of more than 95.0%.

**Table 2:** Performance of the panel in the identification of PCa or subgroups of PCa from normal controls.

Subjects	Frequency (%)	p *	Se (%)	Sp (%)	YI	PPV (%)	NPV (%)	Accuracy (%)
All Pca (*n* = 91)	65 (71.4)	<0.001	71.4	95.8	0.7	95.6	72.6	82.2
AA (*n* = 20)	8 (40.0)	<0.001	40.0	95.8	0.4	72.7	85.2	83.7
CC (*n* = 51)	37 (72.5)	<0.001	72.5	95.8	0.7	92.5	83.1	86.2
HA (*n* = 20)	19 (95.0)	<0.001	95.0	95.8	0.9	86.4	98.6	95.7

PCa, prostate cancer; AA, African American; CC, Caucasian; HA, Hispanic American; Se, sensitivity; Sp, specificity; YI, Youden index; PPV, positive predictive value; NPV, negative predictive value. *, frequencies of the panel in PCa patients were compared with that in normal controls.

## Data Availability

Data are available on request to the corresponding author.
